# Friends, forage, freedom: A cluster analysis investigating horse management styles and welfare in the UK and Ireland

**DOI:** 10.1017/awf.2026.10073

**Published:** 2026-02-24

**Authors:** Wendy Watson, Jill R. D. MacKay, Cathy Dwyer

**Affiliations:** 1 The University of Edinburgh, Royal Dick School of Veterinary Studies, UK; 2 School of Veterinary Medicine and BioSciences, SRUC, UK

**Keywords:** Animal welfare, cluster analysis, friends, forage and freedom, horse health and behaviour, horse management, UK and Ireland

## Abstract

This study aims to improve the welfare and management of recreational horses by identifying how different management styles affect horse health and behaviour. We examined the management styles of recreational horse owners in the UK and Ireland, focusing on social interaction (friends), access to suitable forage (forage), and unrestricted movement (freedom). We collected 1,501 survey responses, distributed via social media, and summarised the characteristics and management choices of the respondents. Using the Divisive ANAlysis cluster package in R, three distinct management styles were identified. The largest differences between clusters were in turn-out, individual stabling, and access to forage. The Horse Centred Management Cluster (HCMC) (n = 956) were more likely to provide their horses with 24-h turn-out and access to a forage source, and interaction with two or more horses. The Combined Management Cluster (CMC) (n = 434) showed a combination of management decisions that differed from the HCMC, including horses being kept in an individual stable for longer periods and being provided with shorter turn-out periods (nine or more hours). The Owner Centred Management Cluster (OCMC) (n = 111) provided a more restrictive management style with a much reduced turn-out time (typically 0–6 h), often with no contact with other horses, and less access to a forage source (0–10 h). We explored associations between management factors (friends, forage, and freedom) and horse welfare-related outputs via owner responses to health and behaviour questions, where behaviour was considered to reflect mental state. The HCMC horses were significantly less likely to exhibit gastrointestinal issues, lameness issues, handling problems, or antisocial behaviours compared to both other groups. This study highlights how management impacts the health and behaviour of recreational horses and can contribute to the development of guidance on improved management and welfare for recreational horses.

## Introduction

Animal welfare research has identified three significant requirements of equine welfare: unrestricted access to forage; movement; and companionship with conspecifics (Phelipon *et al.* 2024). These significant requirements have also been described as the 3Fs or friends, forage and freedom and this expression was believed to have been originally created by equine behaviourist, Lauren Fraser (Phelipon *et al.* 2024). However, the domestic management of the horse often overlooks these basic needs and routinely caters more for owner convenience as opposed to considering the natural behavioural needs of the horse (Goodwin 1999). Horse management often involves individual stabling or restricted movement, i.e., limited turn-out in small areas, sometimes without close contact with conspecifics (Hockenhull & Creighton 2015). Horses may also be fed in ways that does not mimic the pattern of trickle feeding of low-value forage seen in the wild (Ermers *et al.* 2023). Management practices which do not consider the horses’ natural behavioural needs, such as social interaction with conspecifics, continuous provision of a low-quality forage source and unrestricted movement, can result in welfare issues for the horse (McGreevy *et al.* 1995; Goodwin *et al.* 2002; Krueger *et al.* 2021).

Social interaction for horses is often restricted by the housing choices made by their owners, particularly individual stabling (Ruet *et al.* 2019; Schmucker *et al.* 2022). Concerns about lack of opportunity for social interactions have been raised with regard to the amount of time horses spend in individual stabling (Hockenhull & Creighton 2014; Horseman *et al.* 2016), because social isolation can result in stereotypic behaviour, such as box walking or weaving (Lee *et al.* 2011; Krueger *et al.* 2021). Expression of stereotypic behaviour is a widely accepted symptom of stress in domestic and captive animals (Mason 1991) and can be considered a mechanism for stress-coping. Stereotypic behaviour occurs initially where there is motivational frustration, such as lack of social interaction (Cooper *et al.* 2000; Lesimple *et al.* 2019), but can become emancipated from the source of chronic stress through central nervous system dysfunction (McBride & Hemmings 2009). The lack of contact with conspecifics has also been shown as a risk indicator for frustration behaviour in horses, such as increased locomotion in acute situations (e.g. separation from a conspecific), muscle tension, and conflict or displacement behaviours in chronic situations (Pannewitz & Loftus 2023), as well as aggressive behaviours expressed towards people or other animals (Hockenhull & Creighton 2014). Often full contact with conspecifics is not provided for horses due to fear of injury (Hartmann *et al.* 2015), even though horse owners may be aware that group housing would be a preferred choice by the horse (Visser & Van Wijke 2012).

Physiologically, horses have a need for a continual supply of a low-quality forage source or roughage to help prevent gut ulceration and colic (Scantlebury *et al.* 2014; Moore-Colyer 2024). In free-roaming situations, horses spend a large part of the day foraging, dependent on the time of year (Salter & Hudson 1979). In stabled horses, a significant risk of gastric ulcers has been associated with a restricted access to forage for a period of more than 6 h (Luthersson *et al.* 2009).

The lack of access to forage has also been identified as a causal factor in the development of re-directed or stereotypic behaviours (Goodwin *et al.* 2002; Ellis *et al.* 2010; Roberts *et al.* 2017). There is also confusion amongst horse owners with regard to the difference between roughage (fibre) and forage needs, with the horse’s requirements for fibre often being misunderstood. For example, hay cut from lush vegetative pastures is associated with higher levels of protein and energy, and a reduction in fibrous content. Hay cut from pastures with a mature sward has a higher stem-to-leaf ratio and increases the fibre content of the dry matter, which is beneficial for the horse’s roughage requirements and gut health (Ermers *et al.* 2023). Not providing enough roughage or the right type of forage, could be due to the horse owners’ lack of understanding regarding of their animal’s nutritional needs (Hoffman & MSU 2009; Rioja Lang *et al.* 2020). Concerns for obesity, metabolic disease (Furtado *et al.* 2021) and the inability to store large bales of forage have all been suggested as reasons why some horse owners are not meeting their horse’s roughage requirements (Ermers *et al.* 2023).

Individual stabling has been identified as a contributing factor for limited movement in horses, resulting in abnormal locomotor behaviours (Mills & Riezebos 2005), colic due to lack of gut motility (Scantlebury *et al.* 2014) and aggressive behaviour towards people (Hockenhull & Creighton 2015). Associations between oral and ingestive behaviours, such as crib biting, windsucking, wood chewing and eating bedding, have been found in horses being stabled for 21–24 h, during a 24-h period (Hockenhull & Creighton 2015). Hockenhull and Creighton (2014) previously found an association between handling behaviour issues in horses which routinely spent between 12 and 16 h in their stable. Managing horses without stabling may be preferable for the horse’s well-being (Horseman *et al.* 2016), but is not always possible due space restrictions, weather/climate, and pasture destruction. Therefore, horses will often be stabled for at least part of the day (Bradshaw-Wiley & Randle 2023). However, some horse owners may consider individual stabling as the ideal management situation for the horse and may lack understanding of the horse’s need for unrestricted movement (Hockenhull & Furtado 2021).

In a recent study of horses and owners in the UK and Ireland, Watson *et al.* (2025) demonstrated that horse age, breed type, and activity were weakly associated with welfare issues. Approximately one-quarter of the horses in this population were reported by their owners as experiencing lameness issues and another 11% reported their horses had displayed handling issues in the previous month. Additionally, sport horse breed types were twice as likely to have been reported as having gastrointestinal issues, handling issues and three times as likely to exhibit abnormal oral behaviours, compared to other breed types (Watson *et al.* 2025). However, aspects such as the owner’s management strategies in terms of providing access to friends (social interactions), forage and freedom (unrestricted movement and turn-out), as well as their attitudes and knowledge, may be greater contributing factors to horse welfare. The horse’s basic welfare needs are largely the responsibility of the owner/manager and will be affected by their decisions and actions. Horse owner perceptions, attitudes and knowledge have all been shown to affect horse management choices, which consequently affect the well-being of the animal (Visser & Van Wijke 2012; Hockenhull & Creighton 2014; Rioja-Lang *et al.* 2020; Hemsworth *et al.* 2021; Bradshaw-Wiley & Randle 2023).

The first aim of the current study was to investigate if there were clustered horse owner ‘management styles’ in the survey population with regard to the provision of friends, forage and freedom. Secondly, we aimed to determine if associations existed between health and behavioural issues of the horses in the population and those cluster management actions. We hypothesised that owner management choices may cluster around specific approaches to horses’ management. Further, we hypothesised that the management choices of most horse owners would not focus on the horse’s natural behaviour needs of friends, forage and freedom and this would be associated with indicators of poorer welfare.

The outcomes of the cluster analysis, and results of the associations between management styles and health and behaviour issues in the horses, will help to identify specific types of management where horse welfare is impacted. This may provide insight into human behaviours, such as the Dunning Kruger effect, which suggests that humans are not always aware of their lack of knowledge or expertise (Dunning 2011). It is hoped that such human behaviours may be addressed with interventions and instigate further research designed to improve the welfare of horses.

## Materials and methods

### Ethical approval

All research included in this study was approved by the University of Edinburgh’s Human Ethics Review Committee (HERC) HERC_316_19.

### Survey design, participants and recruitment

Data analysed in this paper were collected as described in Watson *et al.* (2025). Briefly, the survey questions were derived using peer-reviewed literature on welfare issues associated with horse management practices. The survey consisted of 60 questions in nine sections containing multiple-choice, fixed-response and Likert-type questions. Some questions allowed the respondents to select multiple answers, whereas other questions were limited to a single response from a list. The survey covered four main categories: owner demographics; horse demographics; horse management; and horse health and behaviour and took between 10–15 min to complete. Respondents were asked to answer the management questions about one specific horse or pony, the one for which they made the majority of management decisions, and subsequently to answer questions for the same animal throughout the survey. The survey was hosted on JiSC (undated) online surveys and was open from January 14th to October 24^th^, 2019. Data on horse and owner demographics were presented in Watson *et al.* (2025), and in the current study we focus upon the responses to management questions related to friends, forage and freedom. A copy of the entire survey can be found in Supplementary material 1.

There were five questions used in our analysis where the category boundaries showed a degree of overlap (Table 1). The questions were radio button, exclusive-response questions, but due to human error the label descriptions themselves overlapped, e.g. Question 10 ‘How many years of experiences do you have with managing horses or ponies?’, a respondent who had managed their horse for 5 years could have chosen the option for ‘2–5 years’ or ‘5–8 years’ correctly. There was no guidance provided to the survey respondents as to which choice would be more appropriate.

**Table 1.** Survey questions with an overlap in category boundaries due to human error in which numerical categories which are not mutually exclusive

Question	Category
Question 10 ‘How many years of experience do you have with managing horses or ponies?’	Less than 2 years 2–5 years 5–8 years 8–14 years 14+years Prefer not to say
Question 22, ‘How long have you managed this horse (regardless of whether or not you own it?)’	2 years or less 2–5 years 5–10 years 10+ years N/A Other
Question 23, ‘How long has your horse or pony been in its current location?’	Less than 6 months 6 months–2 years 2–5 years 5–10 years 10–16 years 16+years
Question 26, ‘What is the approximate age of this horse?’	2 years or less 2–5 years 5–10 years 10–14 years 14–20 years 20+ years
Question 40, ‘During the past week what was the size of the area your horse was (most commonly) turned out on?	Less than an acre 1–2 acres 2–5 acres 5 or more acres Don’t know

It was decided to retain these questions for analysis whilst also acknowledging this as a possible source of noise in the data associated with the boundaries. The rank difference between groups was judged to still be meaningful, as the data are ordinal. For example, Q26, it was unlikely that a 5-year-old horse would be miscategorised as a 20-year-old horse. For Q38 the category for 7–8 h was inadvertently omitted.

### Statistical analysis

#### Cluster analysis

A total of 60 questions were asked in the survey and, of these, 13 focused upon horse management regarding access to friends, forage and freedom (see Table S1; Supplementary material 2). Also used in the current analysis were seven questions regarding general horse owner management attributes, i.e. Q10 the years of experience managing horses, Q13 did the respondents earn an income from equine-related activities and Q30 which asked whether or not respondents insured their horses for veterinary expenses (see Table S2; Supplementary material).

The questions relating to management and friends, forage and freedom were selected *a priori* and a cluster analysis was performed to group these into a ‘management style’. For some of the questions, the answers were regrouped and reduced in number by amalgamating adjacent categories to facilitate analysis. Definitions of the recoded explanatory variables for both the general management and friends, forage and freedom questions are shown in Table 2. A total of seven explanatory variables for the respondent management questions and ten explanatory variables for friends, forage and freedom questions were recoded as a data reduction technique for the cluster analysis in R (Table 2).

**Table 2.** Description of the allocation of responses to new categories for analysis for those questions where there were too many categories for effective Cluster analysis to take place. These were 7 questions about the experience of the humans responding and 10 questions related to their management of their horse. Categories were collapsed into a smaller number by amalgamating adjacent categories. The regrouped categories were used as explanatory variables for the cluster analysis

Survey question	Regrouped
**How many years of experience do you have with managing horses or ponies?**	
Less than 2 years	< 2 years
2–5 years	5 years–14 years
5–8 years	5 years–14 years
8–14 years	5 years–14 years
14+ years	14 years+
**Do you derive an income from equine related activities?**	
Yes, entirely	Yes
Yes, partially	Yes
No not at all	No
**Do you own this horse?**	
Yes, I own this horse	Yes, I own
No, I don’t own this horse	No own
No, I don’t own this horse but I am financially responsible for this horse	No own
No, I don’t own this horse but I have some financial responsibility for this horse	No own
Part loan (I am responsible for the upkeep of the horse)	No own
**How long have you managed this horse (regardless of whether or not you own it?)**	
2 years or less	<2 years
2–5 years	3–10 years
5–10 years	3–10 years
10+ years	10+ years
**How long has your horse or pony been in its current location?**	
Less than 6 months	< 6 months
6 months–2 years	6 months–2 years
2–5 years	2–5 years
5–10 years	5–16 years
10–16 years	5–16 years
16+ years	16+ years
**Do you insure your horse or pony for veterinary expenses?**	
Yes	Yes
No	No
Prefer Not To Say	No
**During past six months have you had your horse shod for any of the following reasons? (Please tick those applicable)**	
Brittle hooves	Remedial shoe
Club foot	Remedial shoe
Cracked hooves	Remedial shoe
For competition	Shod
Laminitic hooves	Remedial shoe
Low heels	Remedial shoe
Navicular syndrome	Remedial shoe
Soft hooves	Remedial shoe
Riding on hard surfaces	Shod
Thin soles	Remedial shoe
I don’t shoe my horse	Barefoot
I always keep my horse shod	Shod
**During the past week if your horse is kept in a stall in a barn how many other horses can it see from its stall?**	
1 other horse	1 or less
2–4	> than 1
5–8	> than 1
+9	> than 1
My horse can’t see any others from its stall	1 or less
I did not keep my horse in a stall at all during the past week	Not kept in stall
**On an average day in the past week, how many other horses can your horse interact with freely (i.e. make physical contact with) with when turned out?**	
No other horses	No other
One other horse only	One only
1–3 horses	2+ horses
4–6 horses	2+ horses
7–9 horses	2+ horses
10+ horses	2+ horses
**On an average day in the past week was your horse most commonly turned out with the same group of horses?**	
Yes	Yes
No	No
Don’t know	D/K
N/A there are no other horses	No
**Forage**	
**During the past week, on average, what type of area was your horse (most commonly) turned out on?**	
Indoor school	Exercise
Outdoor school	Exercise
Paddock (No grass)	Exercise
Pasture – (fertilised, mowed, managed)	Exercise+forage
Pasture – (mowed and managed)	Exercise+forage
Pasture – native (not maintained)	Exercise+forage
My horse is not turned out	Neither
**During the past week, on an average day, which of the following forage sources did your horse (most commonly) have access to? (Please select all of those applicable) n = 10**	
Alfalfa cubes	Processed forage
Chaff	Processed forage
Hay round bale	Hay
Hay square bale	Hay
Haylage	Haylage
Hay pellets	Processed forage
Lucerne (alfalfa)	Processed forage
Pasture grass (Maintained)	Pasture
Pasture grass (Natural)	Pasture
No forage source	No forage source
**During the past week, on an average day, how many hours did your horse (most commonly) have access to a forage source?**	
None	< 3 h
1–3 h	<3 h
4–6 h	4–10 h
7–10 h	4–10 h
11–15 h	11–23 h
16–23 h	11–23 h
24 h	24 h
**Freedom**	
**During the past 3 months what type of stall was your horse kept in for the majority of the time?**	
Loose box – shedrow style	loose
Loose box – American style barn	loose
Loose box housing in an open barn	loose
Loose box continually open to paddock or pasture	Out
Group housing in an open barn	Group
My horse was not kept in a stall during the past 3 months	Out
**During the past week, on average, how many hours (most commonly) was your horse turned out for each day?**	
Not at all	< 1 h
Less than an hour	< 1 h
1–4	1–6 h
5–6 h	1–6 h
9–12 h	9–20 h
13–16 h	9–20 h
17–20 h	9–20 h
Turned out 24 h a day	24 h
**During the past week what was the size of the area your horse was (most commonly) turned out on?**	
Less than an acre	< than acre
1–2 acres	1–2 acres
2–5 acres	2+ acres
5 or more acres	2+ acres
Don’t Know	D/K
**During the past month what type of shelter has been most commonly available for your horse when it was turned out?**	
Hillside	Part Shelter
Man-made shelter	Shelter
Overhang or side of building	Part Shelter
Trees or shrubs	Part Shelter
No shelter	No Shelter

The variables of interest were mainly categorical (e.g. Do you own this horse? Yes/No) and therefore a dissimilarity matrix was used to describe how similar each respondent was to one another using the ‘cluster’ package (Maechler *et al.* 2022) in R Version 3.6.1 (‘Action of the Toes’; the R Foundation 2019). The dissimilarity matrix in R utilises Gower’s distance (Gower 1971) given the number of categorical variables present in the dataset. Gower’s distance is commonly used in cluster analysis to assess the difference between two variables. The distance measured is always a number between 0 (identical) and 1 (maximally dissimilar) and is determined as the average of partial dissimilarities across individuals (Anad 2020).

**Figure 1.**
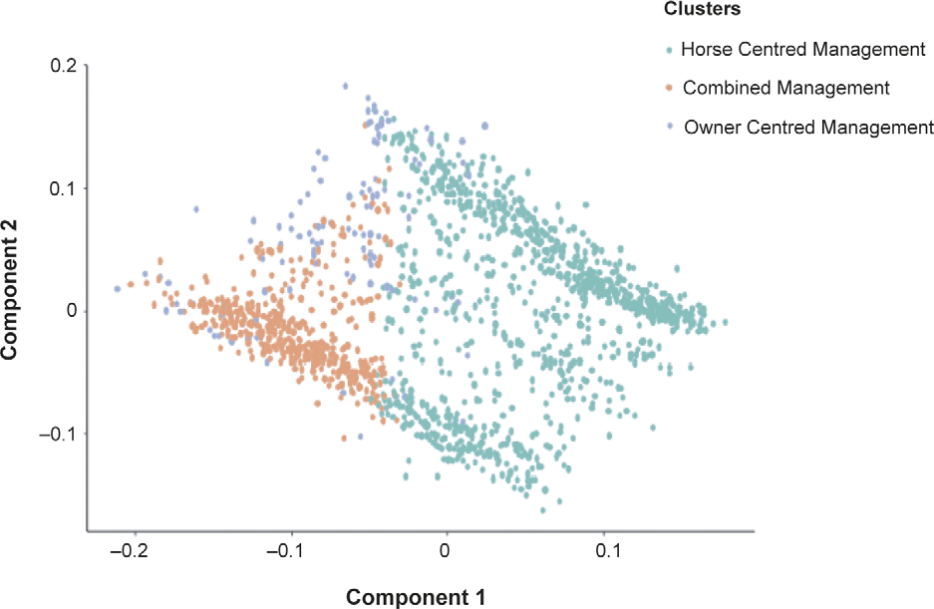
Principal Component Analysis demonstrating the distribution of respondents between Components 1 and 2 for respondents in each cluster (Green = ‘Horse Centred Management Cluster’, Pink = ‘Combined Management Cluster’ and Blue = ‘Owner Centred Management Cluster’) as derived from the cluster analysis in R.

The ‘ape’ package (Paradis & Schliep 2019) was used to perform a Principal Components decomposition on the dissimilarity matrix to correct for negative eigen-values. The DIANA (Divisive ANAlysis Clustering) algorithm from the cluster package was used in R to compute hierarchical clusters based on the dissimilarity matrix and this was visually inspected to explore how many clusters were appropriate (see Figure S1; Supplementary material 2). The within cluster sums of squares were plotted with cluster number to define when the value of adding another cluster did not have a notable effect on the dissimilarity of adjacent data-points, which indicated three clusters as the optimal number to describe the data (Figure 1). No strict criterion was applied to cluster number selection, instead three clusters was considered a pragmatic division which captured a good amount of variation within the data while allowing for simple description (Figure S1; Supplementary material 2).

We chose to describe our management clusters as Horse Centred Management Cluster (HCMC), Combined Management Cluster (CMC) and Owner Centred Management Cluster (OCMC), based on the predominant management styles represented in each cluster. These clusters are also similar to categories described in a thematic analysis by Ross *et al.* (2023).

### Investigation of the impact of management on health and welfare

Specific health and behavioural variables were collapsed into a smaller number of larger categories for analysis as health (four categories): infectious disease; lameness; hoof problems; gastrointestinal issues and behaviour (four categories): abnormal oral behaviour; handling issues; antisocial behaviours; weaving. The grouping and renaming of these owner-reported-related categories is described in Table S3 (see Supplementary material 2). Abnormal oral behaviours included crib-biting, wind-sucking and horses biting and chewing any non-food-related items in ways that were described by the owner as problematic for them. Handling issues included horses perceived to be difficult to manage when being led, groomed, tacked up or for the farrier. Antisocial behaviours included horses being aggressive or threatening aggression towards people or horses including pinning the ears, and lunging at people passing, as perceived to be a problem by the owner.

Pearson Chi-squared tests of independence were conducted in Minitab 18 to investigate if the prevalence of these health and behaviour indicators differed between the clusters. We also included in this analysis whether or not the cluster respondent needed to call out the veterinarian in the past six months. The study utilised a significance level of 0.05, a conventional threshold which represents a Type 1 error, with a 5% risk. Cramer’s V tests (Kim 2017) were used to determine the effect size of the Chi-squared analyses.

## Results

### The larger survey population and their management choices

#### Demographics

Overall, 98% of respondents were female with a median age of 45 years. Fifty-six percent of respondents resided in England, 30% of the population were educated to undergraduate degree level and 50% of the respondents had a total household income of between £20,000–£74,999 before tax (Table S4; Supplementary material 2).

Three-quarters of the survey population did not derive an income from equine-related activities. Most respondents (80%) had 14 or more years of experience with horses and 98% owned and managed the horse they reported on in the survey (Table S4; Supplementary material 2).

#### Management choices of friends, forage and freedom

In the past 3 months, 36% of respondents had not stabled their horses at all but of those that had, the majority (61% of all respondents) had kept their horses in an individual stable. A small proportion (< 2%) had used some form of group housing (Table 3). When considering the prior week to completing the survey, of those horses that were stabled, 11% of respondents said their horse could see only one or no other horses from the stable, whereas most stabled horses could see two or more horses (48%). Very few horses were not turned out at all or for less than an hour each day (1%), and most (51%) of horses were turned out for 24 h a day (Table 3). Nine percent of horses had no interaction with other horses when turned out, whereas 15% were turned out with one other horse only. However, 75% horses were able to socialise with several horses in a group and, of these, the group composition rarely changed (88% horses turned out with the same group).

**Table 3.** Survey responses to questions relating to the management practices which allowed the horse access to friends, forage, and freedom and shoeing and hoof care choices. Values are given as counts and percentage of the total responses (in parentheses) to each question. Total numbers of response are given for each question as not all respondents answered all questions, and some questions (n = 2) allowed multiple answers

Variable	Number of respondents (%)
**Type of stabling n (%) (used in the past 3 months)**	
Not kept in a stable	535 (35.7)
Individual stable (loose box)	920 (61.4)
Group housing	21 (1.4)
⫯Other	22 (1.5)
Total	n = 1,498
**Number of horses seen from stall in the past week**	
Not kept in stable	605 (41.8)
No other horses	31 (2.1)
1 other horse	122 (8.4)
2–4 horses	385 (26.6)
5–8 horses	214 (14.8)
9 plus horses	90 (6.2)
Total	n = 1,447
**Daily turn-out, on average, in the past week**	
Not at all	12 (0.8)
Less than an hour	3 (0.2)
1–4 h	41 (2.7)
5–8 h	143 (9.6)
9–12 h	249 (16.6)
13–16 h	144 (9.6)
17–20 h	140 (9.4)
Turned out 24 h a day	764 (51.1)
Total	n = 1,496
**Free interaction with other horses (an average day in the past week)**	
No other horses	138 (9.2)
Only one other	229 (15.3)
1–3 horses	730 (48.9)
4–6 horses	257 (17.2)
7 or more horses	139 (9.3)
Total	n = 1,493
**Horse turned out with the same group of horses (an average day in the past week)**	
Yes	1,304 (87.5)
No	38 (2.6)
Horse not turned out with others	141 (9.5)
Don’t know	7 (0.5)
Total	n = 1,490
**Forage sources provided on an average day in the past week (multiple choices possible)**	
Pasture (grouped) – maintained or natural	1,353 (44.8)
Hay (grouped) – round bale, square bale	771 (25.6)
Chaff	522 (17.3)
Preserved forage (grouped) – alfalfa cubes, hay pellets, lucerne (alfalfa), haylage	368 (12.2)
Total	n = 3,014
**Hours of access to a forage source on an average day in the past week**	
None	12 (0.8)
1–3 h	16 (1.1)
4–6 h	37 (2.5)
7–10 h	57 (3.8)
11–15 h	102 (6.8)
16–23 h	250 (16.7)
24 h	1,025 (68.4)
Total	n = 1,499
**Size (area) of turn-out on average day in the past week**	
Less than an acre	343 (23.0)
1–2 acres	478 (32.0)
2–5 acres	388 (26.0)
5 or more acres	244 (16.4)
Don’t know	39 (2.6)
Total	n = 1,492
**During the past six months who has most often trimmed your horse’s hooves?**	
Farrier	1,261 (84.1)
Barefoot trimmer	162 (10.8)
Yourself	56 (3.7)
Horse’s feet not trimmed	13 (0.9)
Other	8 (0.5)
Total	n = 1,500
**During past six months have you had your horse shod for any of the following reasons? (multiple choices possible)**	
I always keep my horse shod	584 (31.7)
I don’t shoe my horse	563 (30.5)
Riding on hard surfaces	259 (14.1)
Remedial shoeing	221 (12.0)
For competition	160 (8.7)
Prefer Not to Say	1 (0.1)
¶Other	53 (2.9)
Total	n = 1,841

⫯Open barn in field, grass free paddock ~Grass track system; ¶ Shod in front only, unshod winter, shod in summer.

The most common forage source provided to horses, on an average day in the past week, was either a natural or maintained pasture (45%). Over two-thirds of horses (70%) had 24 h daily access to a forage source, although a small number (4%) had between 0–6 h forage access. Just over half of the respondents (55%) reported that their horses had less than two acres, on average, at turn-out, whereas 16% of horses had more than five acres of turn-out space (Table 3).

Hoof care was provided by a farrier for most respondents (84%) and only 4% provided hoof care themselves. Most horses were shod (67%), with the most common reason being that the horses were always kept shod. Other shoeing reasons were related to activities with the horse, such as for competition reasons (9%) or medical/remedial needs.

### Description of the clusters

**Figure 2.**
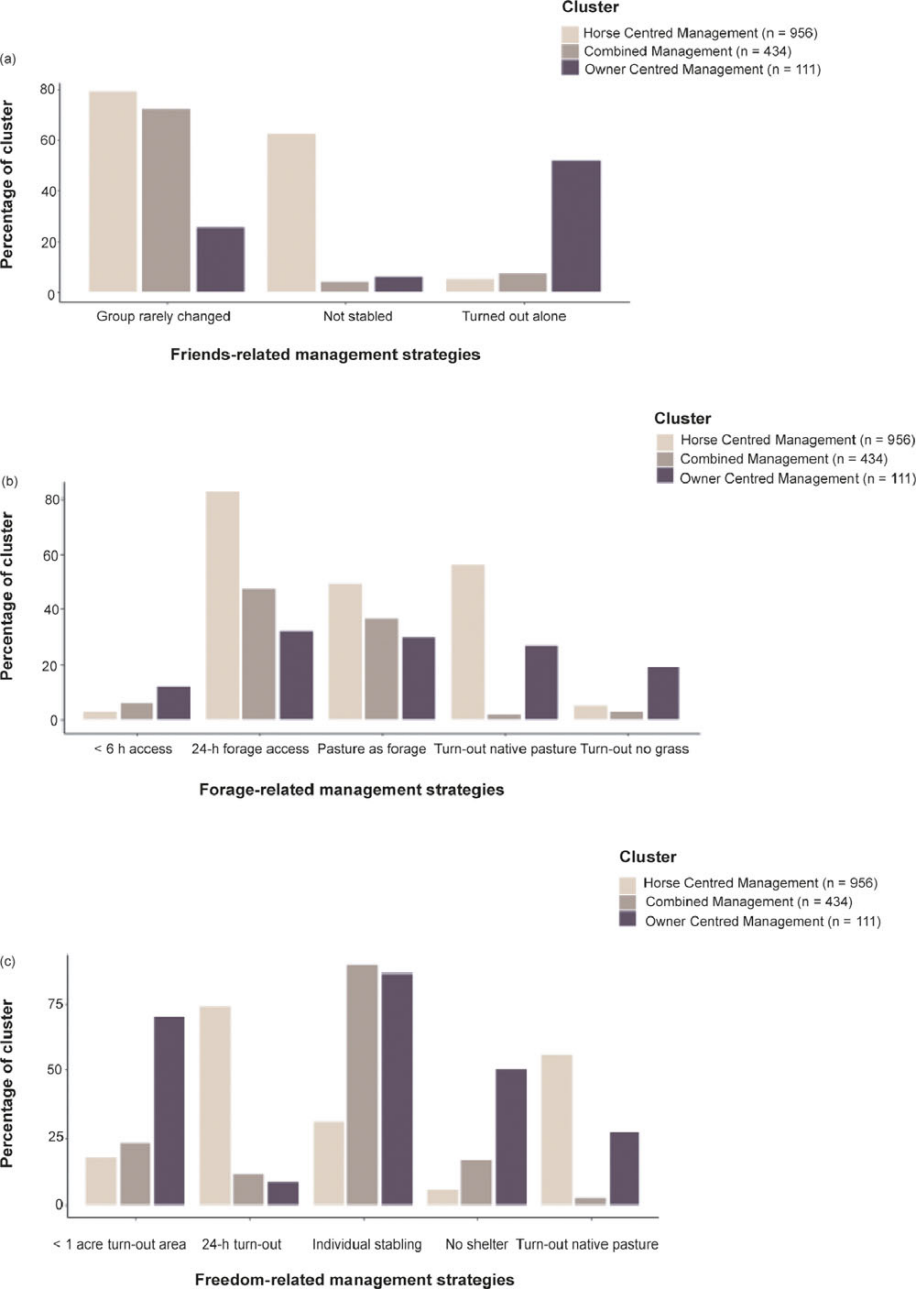
Distribution of responses relating to management questions focused on (a) friends, (b) forage and (c) freedom for the three management clusters shown as Horse Centred Management (light brown), Combined Management (mid-brown) and Owner Centred Management (dark brown). Values are given as the percentage of respondents in each cluster which practised the management strategy.

The key differences between the clusters with respect to the three main areas of friends, forage and freedom are shown in Figure 2 and described below.

#### Horse Centred Management Cluster (HCMC)

The HCMC was typically characterised by horses that were not stabled (59% of respondents’ management in this cluster), had frequent social interactions with two or more horses (80%), grazed on native pastures (56%) with 24 h a day access to a forage source (82%). Horses in this cluster commonly had 24 h daily access to turn-out (74%) and were kept in fields that exceeded two acres (50%) with a variety of different shelters; with trees and shrubs being the most common type of shelter in this cluster (55%) (Figure 2).

#### Controlled Management Cluster (CMC)

The CMC was characterised by horses that were usually stabled individually (89%) but could see one or more horses (92%) from their stable and typically also had free contact with two or more horses when turned out (76%) for 9 h or more per day (68%). The CMC horses had access to managed pastures which were fertilised, mowed and managed (94%), turn-out of less than five acres (62%) and were provided with a forage source for between 11–23 h per day (41%) (Figure 2).

#### Owner Centred Management Cluster (OCMC)

The OCMC was composed of respondents whose horses were usually stabled individually (94%), may not be able to see another horse from their stable (9%) and were also commonly turned out alone (52%). A quarter of the horses managed by the OCMC had 10 h or less access per day to a forage source (25%), on an average day in the past week, and were provided access to managed, mowed and fertilised (39%) or native pastures (27%). Nearly half of the horses in this cluster were turned out for 0–6 h on an average day in the past week (48%), on paddocks of less than one acre (70%) and without access to shelter (50%) (Figure 2).

### Demographic differences between clusters

Approximately 60% of each cluster was represented by English respondents (Table 4). However, twice as many Scottish respondents were in the HCMC (31%) and CMC (28%) compared to the OCMC (14%). In contrast, Irish respondents were twice as likely to be in the OCMC cluster (23%) than in the HCMC (10%) or the CMC (12%) groupings.

**Table 4.** Demographic information of respondents by the three identified clusters ‘Horse Centred Management, ‘Combined Management’ and ‘Owner Centred Management’. Values are counts and percentages of each cluster (rounded to 2 decimal places) in parentheses, unless otherwise stated

	Horse Centred Management Cluster	Combined Management Cluster	Owner Centred Management Cluster
**Number of respondents** (% of total respondents)	956 (63.69%)	434 (28.91%)	111 (7.40%)
**Gender n (%)**			
Female	943 (98.64%)	424 (97.70%)	108 (97.30%)
Male	13 (1.4%)	8 (1.84%)	3 (2.70%)
Prefer Not to Say	0 (0%)	2 (0.46%)	2 (1.80%)
**Country n (%)**			
England/Wales/Isle of Man	569 (59.52%)	259 (59.68%)	70 (63.06%)
Scotland	296 (30.96%)	122 (28.11%)	15 (13.51%)
Ireland/N. Ireland	91 (9.52%)	53 (12.21%)	26 (23.43%)
**Level of Education n (%)**			
No qualification	14 (1.46%)	4 (0.92%)	2 (1.80%)
High school degrees or equivalent	212 (22.18%)	86 (19.82%)	30 (27.03%)
HNC, HND etc	169 (17.68%)	71 (16.36%)	15 (13.51%)
Undergraduate degree	291 (30.44%)	148 (34.10%)	39 (35.14%)
Master’s degree etc.	216 (22.59%)	98 (22.58%)	22 (19.82%)
PhD	38 (3.97%)	21 (4.84%)	0 (0.00%)
Other	16 (1.67%)	6 (1.38%)	3 (2.70%)
**Level of Equine Education n (%)**			
None or Other	590 (61.72%)	248 (57.14%)	68 (61.26%)
BHS Exams	176 (18.41%)	102 (23.50%)	24 (21.62%)
HNC/HND Equine Related	64 (6.69%)	20 (4.61%)	4 (3.60%)
UG Degree Equine Related	79 (8.26%)	41 (9.45%)	5 (4.50%)
PG Degree Equine Related	47 (4.92%)	23 (5.30%)	10 (9.01%)
**Level of Income n (%)**			
<£20,000	96 (10.04%)	34 (7.83)%	11 (9.91%)
£20,000 to £34,999	184 (19.25%)	58 (13.36%)	11 (9.91%)
£35,000 to £49,999	148 (15.48%)	71 (16.36%)	22 (19.82%)
£50,000 to £74,999	161 (16.84%)	77 (17.74%)	24 (21.62%)
£75,000 to £99,999	91 (9.52%)	40 (9.22%)	8 (7.21%)
Over £100,000	76 (7.95%)	55 (12.67%)	10 (9.01%)
Prefer Not to Say	200 (20.92%)	99 (22.81%)	25 (22.52%)

There were no other obvious distinctions between the demographic data and membership of different clusters (Table 4).

### Describing the cluster management styles

Nearly all respondents reported that they were experienced with horse management and more than 70% had 14+ years of experience managing horses. Proportionately, a higher percentage of the OCMC (3%) had less than two years of management experience with horses compared to the HCMC or CMC (each < 1%; Table 5). The OCMC also more commonly reported that they had less than two years managing the horse they reported on in the survey (33%) compared to the HCMC (15%) or CMC (24%). The HCMC (31%) and CMC (22%) were more likely to report keeping their horses in the current location for more than five years compared to the OCMC (16%) (Table 5).

**Table 5.** Responses to questions relating to horse ownership and management by respondents in the three identified clusters of ‘Horse Centred Management, ‘Combined Management’ and ‘Owner Centred Management’. Values are counts and percentage of each cluster (rounded to 2 decimal places) in parentheses

Possible responses	Horse Centred Management Cluster (n = 956)	Combined Management Cluster (n = 434)	Owner Centred Management Cluster (n = 111)
**Question: How many years of experience do you have managing horses?**			
<2 years	6 (0.63%)	2 (0.46%)	3 (2.70%)
5–14 years	167 (17.47%)	95 (21.89%)	27 (24.32%)
14+ years	782 (81.80%)	334 (76.96%)	81 (72.97%)
Prefer Not to Say	1 (0.10%)	3 (0.69%)	0 (0%)
**Question: How long have you managed this horse (reported on in the survey, regardless of whether or not you own it?)**			
<2 years	144 (15.06%)	110 (24.35%)	37 (33.33%)
3–10 years	527 (55.13%)	223 (51.38%)	54 (48.65%)
10+ years	282 (29.50%)	99 (22.81%)	18 (16.22%)
Other	3 (0.31%)	2 (0.46%)	2 (1.8%)
**How long has your horse or pony been in its current location?**			
<6 months	86 (9.0%)	57 (13.13%)	12 (10.81%)
6 months up to 2 years	221 (23.12%)	151 (34.79%)	50 (45.05%)
2–5 years	301 (31.49%)	121 (27.88%)	31 (27.93%)
5+ years	297 (31.07%)	95 (21.89%)	18 (16.22%)
16+ years	49 (5.13%)	10 (2.3%)	0 (0.0%)
Missing	2 (0.21%)	0 (0.0%)	0 (0.0%)
**Do you insure your horse or pony for veterinary expenses?**			
Yes	414 (43.31%)	250 (57.60%)	55 (49.55%)
No	541 (56.59%)	183 (42.17%)	56 (50.45%)
Missing	1 (0.10%)	1 (0.23%)	0 (0.0%)
**During past six months have you had your horse shod for any of the following reasons? (Multiple responses possible)**			
Barefoot	456 (41.12%)	84 (14.21%)	20 (14.29%)
Shod	303 (27.32%)	221 (37.39%)	60 (42.86%)
Remedial shoeing reasons (see reasons- Table 2)	307 (27.68%)	267 (45.18%)	55 (39.29%)
Prefer not to say	1 (0.09%)	0 (0.0%)	0 (0.0%)
¶Other	42 (3.79%)	19 (3.21%)	5 (3.57%)

¶Front only, shod in front only.

A higher percentage of the CMC reported insuring their horses for veterinary expenses (58%) compared to the HCMC (43%) or the OCMC (50%). HCMC respondents were more likely to have unshod horses (41%) compared to OCMC or CMC (14%) (Table 5). Remedial shoeing was more common in the CMC (45%) compared to either HCMC (28%) or OCMC (39%) (Table 5).

### Health and behavioural issues and veterinarian call outs reported in the past six months within the clusters

**Figure 3.**
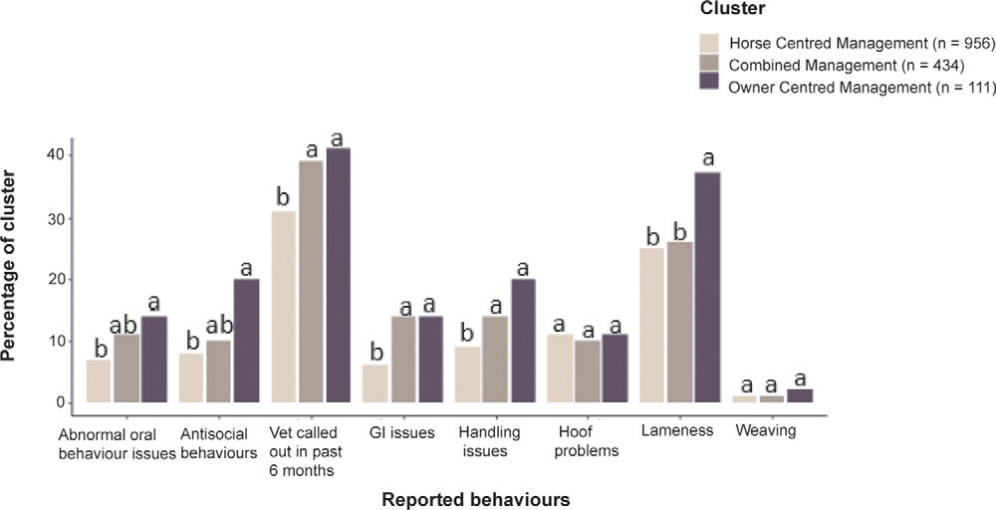
Percentage of cluster respondents who reported horse health and behaviour issues and veterinarian call outs, over the past six months, for Horse Centred Management Cluster (n = 956, light brown), Combined Management Cluster (n = 434, mid brown) and Owner Centred Management Cluster (n = 111, dark brown). Values with differing superscripts in each health/behaviour category differ significantly at *P* < 0.05.

The prevalence of health and behavioural issues, during the past six months, in each cluster is shown in Figure 3. Gastrointestinal issues in horses were significantly less likely to be found in the HCMC, compared to horses in either the CMC or OCMC (Figure 3; Χ^2^_Pearsons_ = 26.54, df = 2; *P* < 0.05, V_Cramer_ = 0.02).

The OCMC were more likely to have reported lameness issues in their horses compared to both the CMC and HCMC (Figure 3; X^2^_Pearsons_ = 7.467, df = 2; *P* < 0.05; V_Cramer_ = 0.01). However, there was no effect of cluster management on the reporting of hoof problems (X^2^_Pearsons_ = 0.303, df = 2; *P* = 0.828, V_Cramer_ = 0.00). Respondents in the HCMC were significantly less likely to have called out the veterinarian, in the past six months, compared with those in the CMC or OCMC (Figure 3; X^2^_Pearsons_ = 12.57, df = 2; *P* < 0.05, V_Cramer_ = 0.01) (Figure 3).

Horses reported to have handling issues, during the past six months, were significantly more common in both CMC and OCMC compared to HCMC (Figure 3; X^2^_Pearsons_ = 16.44, df = 2; *P* < 0.05, V_Cramer_ = 0.01). Similarly, horses displaying abnormal oral behaviours (X^2^_Pearsons_ = 10.25, df = 2; *P* < 0.05, V_Cramer_ = 0.01) and antisocial behaviours (X^2^_Pearsons_ = 13.29, df = 2; *P* < 0.05, V_Cramer_ = 0.01), in the past six months, were significantly more common in the OCMC compared to the HCMC. The incidence of owner-reported abnormal oral behaviour or antisocial behaviour in horses managed in the CMC did not differ from either of the other two clusters. There were no significant impacts of cluster on the reported prevalence of weaving (X^2^_Pearsons_ = 0.142, df = 2; *P* = 0.936, V_Cramer_ = 0.00). All the significant health and behavioural issue results have a weak Cramer V association of between 0.01 and 0.02 (Figure 3).

## Discussion

Friends, forage and freedom are three of the most basic and important behavioural and physiological needs for the welfare of a horse (McGreevy *et al.* 1995; Krueger *et al.* 2021; Phelipon *et al.* 2024). Our study found that many horse owners in the UK and Ireland reported providing their horses with 24-h turn-out, 24-h access to a forage source (mostly pasture) and free interaction with two or more horses for their horse during turn-out, which is a very positive outcome. In this survey population, many horse owners appeared to be providing the behavioural needs of friends, forage and freedom for their horses. Therefore, we reject our hypothesis that the majority of horse owner management choices in our survey would not focus on the horse’s natural behaviour needs. A study in the UK, conducted by Wylie *et al.* (2013), found similar results with only 4% of their population being stabled for 24 h daily and the majority of horses receiving access to pasture and forage. Conversely, a recent study by the Royal Society for the Prevention of Cruelty to Animals (RSPCA; 2025) in England and Wales found that recreational horses were not receiving adequate turn-out or social interaction with other horses. The reasons for these different outcomes are not clear, although it is notable that our study had a high proportion of Scottish respondents, whereas the RSPCA study was focused only on England and Wales. The management style most commonly reported by our respondents, which we described as the Horse Centred Management Cluster (HCMC), was associated with a lower prevalence of health and behavioural issues compared to the Owner Centred Management Cluster (OCMC) or the Combined Management Cluster (CMC). Our results therefore support previous studies which suggest that management which focuses on the behavioural needs of the horse results in a better outcome for the health and welfare of the horses (Bachman *et al.* 2003; Ruet *et al.* 2019; Lesimple 2020; Hall & Kay 2024) and may allow expression of positive welfare states. More emphasis in animal welfare science is currently being focused on the provision of positive welfare for animals, rather than only considering minimising the effect of poor welfare aspects (Vigors & Lawrence 2019). However, there was also evidence reported in our results of some horses usually being stabled, often without sight of another horse, turned out for shorter periods of time, having restricted access to a forage source and often restricted interaction with other horses. In our study, these horses were reported as having more health and behavioural problems than other horses. Thus, although most horse owners in this survey population reported providing good options for friends, forage and freedom for their horses, the survey also uncovered that some horses did not have sufficient access to friends, forage and freedom.

Relatively few studies have focused on cluster analysis and horse management. A previous cluster analysis study (Visser & Van Wijke Janssen 2012) showed distinct groups of horse enthusiasts in a European population with varying levels of equine knowledge and expertise. In our study, a very high percentage of the respondents, across all clusters, had 14 or more years of experience managing horses and could therefore be considered to be very experienced horse managers. However, higher percentages in the CMC and the OCMC reported that they had managed the horse reported in the survey for two years or less compared to HCMC respondents. Although these respondents may have been experienced owners, they may have used a different management strategy for a new horse or may have been more likely to own individual horses for shorter periods of time than the HCMC group.

Respondents in the HCMC more commonly reported providing access to the 3Fs (friends, forage and freedom) than respondents in the other two clusters. HCMC respondents were also significantly less likely to have called out the veterinarian in the past six months. This was associated with significantly fewer health or behavioural issues reported in the HCMC cluster’s horses. This may be because these owners genuinely had fewer issues with health or behaviour of their horses. However, it could potentially be because of reduced close-up observation of the HCMC horses, or less handling of their horses, in a population of horses spending more time turned out. HCMC respondents may have prioritised a more ‘natural’ approach to horse management, which may have reduced the likelihood of them observing health issues in their horses. The HCMC cluster respondents were also the least likely cluster to insure their horses for veterinary treatment. This may reflect a more relaxed approach to horse management and a differing approach to risk compared to respondents in different clusters, or an assumption that horses managed ‘naturally’ are less likely to need to see a veterinarian. Alternatively, previous research suggests that horse owners who competed with their horses were more likely to insure their animals for veterinary expenses, compared to those owners who did not compete (Stowe *et al.* 2022). However, the reasons behind the HCMC reported choices for veterinary insurance are not clear.

The reasons for shoeing were diverse in the survey population. A higher percentage of the HCMC chose to keep their horses barefoot, in comparison with the other two clusters, whereas almost half of the CMC reported shoeing their horses for remedial reasons. Although there may be some associations between poor shoeing practices and musculoskeletal injuries (Lesimple 2020), remedial shoeing can also support recovery from injury or disease, thus links to welfare are complex. Relatively little is known and understood regarding the hoof management strategies of the UK horse-owning population and further investigation is needed to understand the reasons behind hoof management choices.

### The cluster analysis – differences in management styles

Few demographic differences were apparent between the cluster respondents except that, proportionately, a greater percentage of Scottish respondents tended to be in the HCMC and CMC, rather than the OCMC, whereas proportionately more Irish respondents were in the OCMC than in the other clusters. Having a higher number of Scottish respondents in the clusters may have affected the results with respect to the numbers of respondents who were providing the 3Fs for their horses. This is further discussed later in ‘forage’ cluster management, regarding access to rough grazing land.

A higher percentage of the HCMC reported keeping their horses in the current location for more than two years, compared to the CMC and the OCMC, and had managed these horses for longer. Hockenhull and Creighton (2014) found that a reduced length of time horses had been in the same place was associated with handling issues. In our study, horses managed by the HCMC, who were more likely to keep their horses in the same location for longer than the other clusters, had significantly fewer handling issues than the horses managed by the CMC or OCMC. However, because more of the horses in the CMC and OCMC were reported to have been managed for two years or less, they could not have kept their horses in the current location for more than two years, and this may be confounded with duration of horse management.

### Management differences between the clusters relating to ‘friends’

Access to conspecifics is considered a fundamental aspect of positive welfare for horses and is suggested to confer greater welfare importance than either forage or freedom (Zeitler-Feicht *et al.* 2024). The survey responses revealed that 9% of horses could not freely interact with another horse on an average day in the past week and a higher percentage of horses managed by the OCMC were less likely to have social access with any other horses. Restricted access to conspecifics is associated with higher levels of stress in horses (Krueger *et al.* 2021) and our results suggest that some horses reported in our survey may have experienced stress associated with social isolation. Additionally, if the horses in the OCMC were provided with contact with others, they were more commonly not turned out with the same group of horses compared to other clusters. There is an increased likelihood of frustrated behavioural problems for horses who are turned out into a varied group of horses (Hockenhull & Creighton 2014). This may explain why horses managed by the OCMC were significantly more likely to display handling issues, abnormal oral behaviours and antisocial behaviours, compared to the HCMC. Therefore, these behaviours could be due to the restricted opportunities that these horses were provided for social contact.

One frequently cited reason for a lack of turn-out with conspecifics is a fear of injury, (Henderson 2007; Hartmann *et al.* 2015; Phelipon *et al.* 2024). The risk of injury could also be another reason why only a very small percentage of respondents provided group housing in our study. In previous studies (Visser & Van Wijke Janssen 2012; Hartmann *et al.* 2015), horse enthusiasts agreed that horses may prefer to be housed in a group situation, even though this was not their own horse’s management, with the majority providing individual stabling for their horses due to fear of injury. However, there can be less risk of injury for keeping horses in groups when there is sufficient space for unrestricted movement, ample opportunities for foraging, and the potential for positive social interactions (van Dierendonck 2006). Dissemination of education on this topic may be beneficial for some horse owners/managers as it could be helpful in encouraging less restrictive social interaction opportunities for horses (Hartmann *et al.* 2015).

Another reason for not providing group housing could be because it is not an available option. The RSPCA ‘Horse Sense’ report (2025) suggests that a major issue for equine welfare in England and Wales is a lack of equine-keeping facilities with adequate access to turn-out, grazing and socialisation. Traditionally, in the UK, horses are housed in single stables, with three full walls and a front wall with a half door (Robertson *et al.* 2024), with some limited ability to see other horses. Group housing of horses was uncommon in our study and even if respondents wished to keep their horses in this type of housing, it may be difficult to find such accommodation on many livery yards.

### Management differences between the clusters relating to ‘forage’

Most of the respondents in the survey reported that they provided natural pasture as a forage source for a 24-h period. A horse’s physiological need for a constant source of low-quality high fibre forage is important for their health and well-being (Ermers *et al.* 2023). Our study found that a higher percentage of HCMC respondents provided access to a natural pasture source for 24 h compared to the other clusters. The CMC and OCMC managers more commonly provided a managed pasture source (i.e. fertilised, mowed, managed) for their horses. Possible reasons for being able to provide a natural pasture source could be geographical, as there was a higher proportion of HCMC and CMC respondents based in Scotland. Scotland has 58% rough grazing compared to England with only 9% (Scottish Agricultural Report 2016). Providing access to pasture is important for the horse’s digestive well-being, but providing access to the wrong type of pasture such as that which is provided for production animals, combined with an overabundance of grazing can lead to laminitis and metabolic syndrome for some horses, particularly native breeds and ponies (Harris *et al.* 2006; Bott *et al.* 2013; Furtado *et al.* 2022). A higher percentage of the OCMC horses were turned out in a paddock with no grass or an outdoor school and more owners also reported that they only provided access to a forage source for up to 6 h per day compared to other clusters. Lack of access to forage produces a reduction in salivation, which compromises the buffering effect on gastric acidity. However, horses may still want to express foraging behaviour, which can result in abnormal oral behavioural issues such as crib-biting and wood chewing (Waters 2002). Proportionally, a higher percentage of horses managed by the OCMC exhibited abnormal oral behaviours compared to the other clusters. Restricted access to forage also often results in gastrointestinal (GI) issues (Scantlebury *et al.* 2014; Moore-Colyer 2024) and GI issues were significantly less likely to be found in horses managed by HCMC respondents, compared to horses in both the CMC and OCMC.

Restriction of pasture as a forage source may relate to seasonal considerations during the winter due to potential damage to the land and/or livery restrictions (Furtado *et al.* 2022). However, a restriction of a forage source for less than 6 h and turn-out in a paddock with no grass, by the OCMC respondents, may have been linked with a concern for obesity or metabolic disease (Furtado 2019; Cameron *et al.* 2021; Ross *et al.* 2023; Naydani & Coombs 2025). Alternatively, Ross *et al.* (2024) suggested that horse owner management regarding obesity has an emotional connection, with some owners in their study reporting that they were conflicted about the animal’s mental well-being and implementing weight management strategies. Ross *et al.* (2023) also highlighted that some of the owners in their research restricted turn-out during the summer period for several reasons including weight control, manure management and to alleviate contact with insects. Forage may also be substituted with energy-dense feed for performance horses, leading to a decrease in the proportion of the diet derived from forage (Harris *et al.* 2017).

### Management differences between the clusters relating to ‘freedom’

Responses to the questions relating to ‘freedom’ provided the strongest differentiation between the clusters. A higher percentage of the HCMC horses were reported to have been turned out for 24 h, and not kept in an individual stable, compared to horses managed by CMC and OCMC respondents. OCMC respondents more commonly provided access to small paddocks with no shelter, compared to the other clusters, which suggests that a lack of space could perhaps be one of the reasons behind less time for turn-out. Horses managed at livery have been reported as being less likely to be able to self-exercise (i.e. less unrestricted movement during turn-out) than owners who kept their horses at home (Naydani & Coombs 2025). The OCMC horses were also significantly more likely to have been reported as having lameness issues which has also been linked to lack of turn-out (Reilly & Byrk-Lucy 2021).

Hockenhull and Creighton (2014) found that that there was an increase in handling issues and aggressive behaviour towards people in horses kept in their stables for 13–16 h. In our results, the OCMC and CMC horses, which were reported to have been turned out for shorter periods on an average day in the past week, were also more commonly reported as having handling issues, compared to the HCMC horses. As mentioned above, possible reasons for the lack of time for turn-out or restriction of turn-out due to area could have been because of seasonal reasons, such as preservation of grass in the winter or during periods of wet weather (Longland 2013). However, this survey was conducted through the winter and summer periods, asking respondents to report on the previous three months, so this is unlikely to explain all the variation in access to grazing. Other reasons include livery restrictions or restrictions due to an overabundance of grazing and concerns about health-related issues such as laminitis, metabolic disease and obesity (Furtado 2019; Furtado *et al.* 2022). Turn-out is also strongly linked to social interaction and access to forage, and it is sometimes difficult to separate all three of these natural behavioural needs in horses to understand the stressors caused by insufficient access to each (Krueger *et al.* 2021).

### Study limitations

The management aspects of friends, forage and freedom are often intractably interlinked, and it is challenging to separate the nuances and effects of each category. For example, turn-out was commonly provided on pasture, which is also a source of forage and an opportunity for interaction with conspecifics. Although we asked for specific time-frames in the management questions related to friends, forage and freedom, we did not ask for information on specific seasonal variations, which could have affected the outcomes. Access to pasture, for example, can be affected by seasonal variations, with fears of destruction of the land playing a factor in the turn-out of horses to pasture in the winter (Longland 2013; Furtado *et al.* 2022).

Comparison of percentages of categorical data across clusters is an effective way to understand how clusters differ from each other by providing clarity and helps in distinguishing cluster characteristics (Halkidi *et al.* 2001). The Cramer’s V tests clarified the strength of the relationships between categorical variables from Chi-squared analysis (Kim 2017) and although there were significant differences between the clusters and the health and behavioural issues, the Cramer’s V suggested weak relationships between these variables and the differences discussed regarding these data should be considered cautiously. As the results were all owner-reported, and not professional opinions, caution in interpretation should also be considered with regard to the significance of the health and behavioural results. The respondents may also have provided answers which they may have felt were the ‘right answers’, rather than true answers. We note that respondents who provided better welfare for their horses may have been more likely to respond to the survey, which also may have skewed the results. Further discussion of the study design and survey methodology can be found in Watson *et al.* (2025).

### Animal welfare implications

This study reports large numbers of horse owners were turning their horses out for 24 h daily, providing their horses with a forage source for 24 h and allowing their horses to interact with 1–3 horses when turned out. This suggests that, for most respondents in our study, the horses’ basic behavioural needs were being considered which is encouraging information. There also appeared to be a relatively low prevalence of horse health and behavioural issues in this population. This also suggests a positive outcome in that owners appear to be largely meeting the behavioural needs of horses in this survey population. The results suggest that there were three distinctive styles of horse managers in the study population and that, although small in numbers, some of the managers did not provide the basic behavioural needs for their horses. This also appeared to be associated with an increased probability of poorer welfare outcomes (health and behaviour) in the horses managed in this way. There may be multiple factors that could have affected access to friends, forage and freedom for horses reported in our study, including owner characteristics and preferences as well as the opportunities or restrictions that owners may have in terms of space or livery provision. A more detailed understanding of the constraints and opportunities to provide the 3Fs for all horses is needed to help develop effective interventions.

## Conclusion

Respondents within the HCMC cluster were significantly more likely to provide their horses with the three key welfare factors of friends, forage, and freedom. This was indicated by regular interaction with two or more conspecifics, access to native pasture, and 24-h turn-out. Horses managed under this style were also significantly less likely to have required veterinary attention in the past six months and were less likely to have been reported as exhibiting GI issues, handling issues, abnormal oral behaviours, or antisocial behaviours within the same time-frame. These findings suggest a positive association between the HCMC management approach and improved equine welfare outcomes.

In contrast, horses managed under the OCMC approach were confined to stabling for extended periods, had less access to forage and sometimes provided limited or no physical interaction with other horses during turn-out. OCMC horses also demonstrated significantly higher reported rates of GI issues, lameness issues, handling difficulties, abnormal oral behaviours, and antisocial behaviours over the past six months, supporting an association between this management style and reduced welfare outcomes in this cluster.

Whilst the overall number of horses exhibiting poor welfare indicators in this study was relatively small, these findings underscore the importance of further in-depth investigation. Qualitative research methods, such as interviews, may be valuable in explaining the underlying motivations behind owners’ decisions to limit horses’ access to friends, forage, and freedom. The identification of different management styles which do consider the behavioural needs of the horse, could also provide further insight for investigation of the potential barriers to/or opportunities for managing horses in ways which are beneficial to their health and well-being.

## Supporting information

10.1017/awf.2026.10073.sm001 Watson et al. supplementary material 1 Watson et al. supplementary material

10.1017/awf.2026.10073.sm002 Watson et al. supplementary material 2 Watson et al. supplementary material

## Supplementary material

The supplementary material for this article can be found at http://doi.org/10.1017/awf.2026.10073.
