# Supplementary material for: Friends, forage, freedom: A cluster analysis investigating horse management styles and welfare in the UK and Ireland
Source: Anim Welf. 2026 Feb 24;35:e15. doi: 10.1017/awf.2026.10073 (PMC12936806; doi:10.1017/awf.2026.10073)
Supplement: Watson et al. supplementary material 1 — Watson et al. supplementary material [file S0962728626100736sup001.pdf]

Horses in the U.K. and Ireland

Page 1: Privacy Notice

THE UNIVERSITY of EDINBURGH
The Royal (Dick) School
of Veterinary Studies

The Jeanne Marchig International Centre for Animal Welfare Education

Hello and welcome to this survey about equine management.

This survey is part of a PhD research project investigating the current health and management of horses and ponies in the U.K. and Ireland.

The data collected here will be used to form part of the PhD, and some of the data may be used in other ethically approved research. Anonymised data may be reported at conferences or in research papers to help other stakeholders learn from our experience.

This data **will not** be used to identify you as an individual. It will take approximately 10-15 mintues to complete.

If you do not wish to take part, you can close this browser window now. If you would like to know more about how we will use your data, you can read the statement below.

1. Please tick to confirm you agree to participate in the interviews

- [ ] I agree

Why are we running this survey?

We want to know how people in the U.K. and Ireland currently manage their horses and ponies in order to gain a more detailed insight into current horse keeping practices.

Who is responsible for this survey?

Wendy Watson BSc.(Hons), MSc. part-time PhD student at the University of Edinburgh, is responsible for this survey.

This survey is being managed by Wendy Watson email: UofEdEquinePhD@ed.ac.uk

Who will have access to my responses?

- Wendy Watson, who is the primary researcher, as well as the members of her research team as listed below.
- Professor Cathy Dwyer, Dr. Jill MacKay, Dr. Patrick J. Pollock, Royal (Dick) School of Veterinary Studies, University of Edinburgh

What if I decide I want to withdraw my response?

We are not collecting any identifying information in this survey and so are unable to withdraw individual responses after you have submitted your survey. Before you have completed the survey, we will not save any data, and so you may choose to stop the survey at any point before the end without submitting your data.

When will be data be destroyed?

The data will be destroyed 3 years post the end of the evaluation project (January 2027).

If you wish to continue please tick the box below to confirm you agree to participate in the survey. Many thanks for your time.

2. Please tick to confirm you agree to participate in the survey ✱ *Required*

○ I agree

Page 2: Section 1 - About You

3. I define myself as being:

- Female
- Male
- Prefer Not To Say
- In Another Way

4. Your age in years is (please just insert number):

5. What is the first section of your postcode e.g. EH25

6. What is the first section of the postcode where you keep your horse e.g. EH25

7. What is the highest educational qualification you have obtained? If you are currently enrolled in an educational programme please indicate the highest qualification you have **received**.

- No qualification

- High school degrees or equivalent e.g. GCSEs/National 5s/Standard Grades/Highers/ A-Levels/Leaving Certificate)
- Modern Apprenticeship/Higher National Certificate/ Higher National Diploma
- Bachelors / Ordinary Degree / Graduate Diploma Honours Degree
- Masters Degree / Postgraduate Diploma or Certificate
- PhD
- Other

7.a. If you selected Other, please specify:

8. Do you have any of the following equine qualifications? (Please tick all those appropriate to you)

- HNC equine related
- HND equine related
- British Horse Society (BHS) Exams
- Undergraduate Degree - equine related
- Postgraduate Degree - equine related
- None
- Other

8.a. If you selected Other, please specify:

9. If you selected BHS Exams in the previous question please let us know the highest level you have achieved:

10. How many years of experience do you have with managing horses or ponies?

- Less than 2 years
- 2-5 years
- 5-8 years
- 8-14 years
- 14+years
- Prefer not to say

11. Which of the following equine related organisations are you **currently** a member of?

- British Horse Society
- Breed Organisation
- Pony Club
- Riding Club
- None
- Other

11.a. If you selected Other, please specify:

11.b. Which of the following equine related organisations have you **previously** been a member of?

- [ ] British Horse Society
- [ ] Breed Organisation
- [ ] Pony Club
- [ ] Riding Club
- [ ] None
- [ ] Other

11.b.i. If you selected Other, please specify:

12. If you wish, please share the salary range for your **total household** income per annum **before** tax.

- Less than £20,000
- £20,000 to £34,999
- £35,000 to £49,999
- £50,000 to £74,999
- £75,000 to £99,999
- Over £100,000
- Prefer not to say

13. Do you derive an income from equine related activities?

- Yes, entirely
- Yes, partially
- No not at all
- Prefer not to say

Page 3: Section 2 - Horses or Ponies Under Your Management

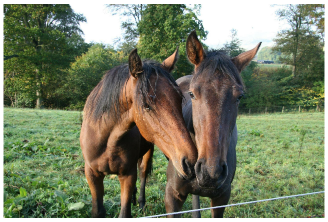

In this survey, we are interested in how horses and ponies are managed.

By 'manage' we mean what choices are made about feeding, housing, healthcare and turn-out of the animal. You can manage a horse or pony that you don't own, for example if you have a part-loan, but you can also own a horse or pony and have someone else manage it.

14. Do you currently manage (i.e. make the majority of decisions about feeding, housing, etc.) a horse or pony?

- Yes
- No
- Don't Know

15. How many horses or ponies do you currently manage? (Just the number please i.e.0=none or 1,2,3 etc)

16. How many other equids do you currently manage? (Just the number please i.e.0=none or 1,2,3 etc)

17. What are these equids?

- [ ] Donkey
- [ ] Mule
- [ ] Other
- [ ] N/A

18. Do you currently own a horse, pony, or other equid (even if you do not make the choices about management?)

- ( ) Yes
- ( ) No
- ( ) Don't Know

Page 4: Section 3 - About Your Horse or Pony

For the rest of this survey we would like you to answer the questions about **one specific horse or pony**. Please answer for the **same animal** throughout.

Ideally, this should be an animal for whom you make the majority of management decisions about.

(In the questions we will say 'horse' but it is okay to answer for a pony)

19. What is the name of the horse or pony which you are referring to for this survey?

20. What type of equid is the animal?

- Horse
- Pony (under 14.2 hh)
- Other

20.a. If you selected Other, please specify:

21. Do you own this horse?

- Yes I own this horse
- No I don't own this horse
- No, I don't own this horse but I am financially responsible for this horse

- No, I don't own this horse but I have some financial responsibility for this horse
- Part loan (I am responsible for the upkeep of the horse)
- N/A
- Other

22. How long have you managed this horse (regardless of whether or not you own it?)

- 2 years or less
- 2 - 5 years
- 5 -10 years
- 10+ years
- N/A
- Other

22.a. If you selected Other, please specify:

23. How long has your horse or pony been in its current location?

- Less than 6 months
- 6 months – 2 years
- 2-5 years
- 5 -10 years
- 10 -16 years
- 16+years

24. On average, how often do you see your horse or pony each week?

- ◯ Once or twice per week
- ◯ Three to five times per week
- ◯ Once every day
- ◯ Several times per day
- ◯ Less than once per week
- ◯ Not at all

Page 5: Section 4 - Your Horse or Pony

25. What type of horse or pony do you own/manage?

- Arab type
- Cob type
- Crossbreed
- Draft horse
- Hunter type
- Irish Draught
- Minature horse
- Mountain and Moorland
- Sport horse type
- Thoroughbred type
- Warmblood
- Western type
- Other

25.a. If you selected Other, please specify:

26. What is the approximate age of this horse?

- 2 years or less
- 2-5 years
- 5-10 years
- 10-14 years
- 14-20 years
- 20+ years

27. What is your horse’s sex?

- Intact stallion
- Mare
- Gelding

- Don't Know

28. What is the approximate height of your horse?

- 12hh and under
- 12.1-14.2
- 14.3-15.1hh
- 15.2-16hh
- 16.1-17hh
- 17hh and over

29. During the **past month** which of the following activities listed below have you engaged in with your horse?: (Please tick all that apply to you and your horse):

- [ ] Breeding of your horse or pony
- [ ] Dressage (Unaffiliated)
- [ ] Dressage (Affiliated)
- [ ] Driving (Unaffiliated)
- [ ] Driving (Affiliated)
- [ ] Endurance (Unaffiliated)
- [ ] Endurance (Affiliated)
- [ ] Eventing (Unaffiliated)
- [ ] Eventing (Affiliated)
- [ ] Hacking
- [ ] Hand walking
- [ ] Hunting
- [ ] Lunging
- [ ] Mounted games
- [ ] Ponying (horse being led along with a ridden horse)

- ☐ Round pen work
- ☐ Show jumping (Unaffiliated)
- ☐ Showjumping (Affiliated)
- ☐ Structured schooling (Work on the flat)
- ☐ Structured schooling (jump work)
- ☐ TREC
- ☐ Use of a treadmill
- ☐ Use of a hotwalker
- ☐ Western riding
- ☐ Vaulting
- ☐ Showing in hand
- ☐ Showing (General)
- ☐ None of the above
- ☐ Other

29.a. If you selected Other, please specify:

____

30. Do you insure your horse or pony for vet expenses?

- ○ Yes
- ○ No
- ○ Prefer Not To Say

Page 6: Section 5 - Management of Your Horse or Pony

Stabling

31. During the **past 3 months** what type of stall was your horse kept in for the majority of the time?

- Loose box - shedrow style
- Loose box - American style barn
- Loose box housing in an open barn
- Loose box continually open to paddock or pasture
- Group housing in an open barn
- My horse was not kept in a stall during the past 3 months
- Other

31.a. If you selected Other, please specify:

32. During the **past week** if your horse is kept in a stall in a barn how many other horses can it see from its stall?

- 1 other horse
- 2-4
- 5-8
- +9
- My horse can't see any others from its stall
- I did not keep my horse in a stall at all during the past week

33. During the **past month** please indicate which items you have used in your horse's stall (Please select all of those appropriate):

- ☐ Anti-weaving grill (v-shaped grille attached to the stall door)
- ☐ Anti-wood chewing covering (metal covering on the horizontal surfaces in the stall)
- ☐ Anti wood chewing covering (carpet or rubber on horizontal surfaces in the stall)
- ☐ Anti-wood chewing topical preparation
- ☐ Automatic waterer
- ☐ Corner feeder
- ☐ Food related toy
- ☐ Hay net
- ☐ Hay rack (wall mounted)
- ☐ Puzzle ball
- ☐ Salt lick
- ☐ Self brushing equipment
- ☐ Stable mirror
- ☐ Toys
- ☐ Water bucket
- ☐ Other
- ☐ N/A I don't keep my horse in a stable

33.a. If you selected Other, please specify:

34. During the **past week** what type of bedding has been used in your horse's stall? (Please select all of those appropriate):

- ☐ Peat
- ☐ Rubber mats or mattress with another type of bedding
- ☐ Rubber mats alone

- ☐ Rubber 'mattress type' mats alone
- ☐ Shredded paper
- ☐ Straw
- ☐ Wood shavings
- ☐ Wood sawdust
- ☐ Wood pellets
- ☐ My horse is not stabled
- ☐ Other

34.a. If you selected Other, please specify:

35. During the **past month** please tell us how your horse's coat has been managed. (Please select all of those appropriate):

- ☐ Brushing 2 or 3 times per week
- ☐ Daily brushing
- ☐ Weekly brushing
- ☐ No brushing during the past month
- ☐ Fully clipped
- ☐ No clipping during the past month
- ☐ Partially clipped
- ☐ Other

35.a. If you selected Other, please specify:

36. During the **past winter months, from Dec-Mar,** please tell us what rugging you have most often provided for your horse when it is **in its stable**:

- Layered rugs
- Lightweight rug (0 up to 200g fill)
- Medium weight rug (200+ to 300g fill)
- Heavyweight rug (over 300g fill)
- No rug on in stables
- My horse was not stabled during the past winter months Dec-Mar
- Other

36.a. If you selected Other, please specify:

Turnout for Your Horse or Pony

37. During the **past winter months, from Dec-Mar**, please tell us what rugging you have used **most often** for your horse when it is **turned out:**

- Lightweight rug (Up to 200g fill)
- Medium weight rug (200+ to 300g fill)
- Heavyweight rug (over 300g fill)
- Layered rugs
- No rug during the past winter months from Dec-Mar
- Other

37.a. If you selected Other, please specify:

38. During the **past week, on average,** how **many hours** (most commonly) was your horse **turned out** for **each day**?

- ◯ Not at all
- ◯ Less than an hour
- ◯ 1-4 hours
- ◯ 5-6 hours
- ◯ 9-12 hours
- ◯ 13-16 hours
- ◯ 17 -20 hours
- ◯ Turned out 24 hours a day

39. During the **past week, on average,** what type of area was your horse (most commonly) **turned out** on?

- ☐ Indoor school
- ☐ Outdoor school
- ☐ Paddock (No grass)
- ☐ Pasture - (fertilised, mowed, managed)
- ☐ Pasture - (mowed and managed)
- ☐ Pasture - native (not maintained)
- ☐ My horse is not turned out
- ☐ Other

39.a. If you selected Other, please specify:

40. During the **past week** what was the size of the area your horse was

(most commonly) **turned out** on?

- Less than an acre
- 1-2 acres
- 2-5 acres
- 5 or more acres
- Don't know

41. During the **past month** what type of shelter has been most commonly available for your horse when it was **turned out**?

- Hillside
- Man-made shelter
- No shelter
- Overhang or side of building
- Trees or shrubs
- Other

41.a. If you selected Other, please specify:

42. On an **average day in the past week**, how many other horses can your horse interact with freely (i.e. make physical contact with) with when **turned out**?

- One other horse only
- No other horses
- 1-3 horses
- 4-6 horses

- 7-9 horses
- 10+ horses

43. On an **average day in the past week** was your horse most commonly **turned out** with the same group of horses?

- Yes
- No
- N/A there are no other horses
- Don’t know

44. How often does the group of horses turned out with your horse change?

- Never
- Rarely
- Occasionally
- Often
- My horse is not turned out in a group

45. During the **past month** were there any other animals turned out with your horse?

- Alpaca
- Cattle
- Goats
- Llama
- Sheep
- No other animals

- [ ] Other

45.a. If you selected Other, please specify:

Page 7: Section 6 - Nutrition for Your Horse or Pony

46. During the **past week, on an average day,** which of the following forage sources did your horse (most commonly) have access to? (Please select all of those applicable)

- ☐ Alfalfa cubes
- ☐ Chaff
- ☐ Hay round bale
- ☐ Hay square bale
- ☐ Haylage
- ☐ Hay pellets
- ☐ Lucerne (alfalfa)
- ☐ No forage source
- ☐ Pasture grass (Maintained)
- ☐ Pasture grass (Natural)
- ☐ Other

46.a. If you selected Other, please specify:

____

47. During **the past week, on an average day**, how many **hours** did your horse (most commonly) have access to a forage source?

- ☐ None
- ☐ 1-3 hours
- ☐ 4-6 hours
- ☐ 7-10 hours
- ☐ 11-15 hours
- ☐ 16-23hrs

- [ ] 24 hours

48. During **the past week, on an average day**, how many concentrated or hard feed meals did your horse consume?

- ( ) 1 meal per day
- ( ) 2 meals per day
- ( ) 3 meals per day
- ( ) 4 or more meals a day
- ( ) No concentrate/hard feed meals
- ( ) Don't know

49. During the **past week, on an average day,** which of the following supplements were (most commonly) provided for your horse? (Please select all of those applicable):

- [ ] Calming supplement
- [ ] Coat enhancing supplement
- [ ] Digestive supplement
- [ ] Forage balancer
- [ ] Hoof supplement
- [ ] Oil -vegetable, coconut etc.
- [ ] Fish oil
- [ ] Respiratory supplement
- [ ] Salt or mineral lick
- [ ] Salt or mineral (loose)
- [ ] Topline/muscle gain supplement
- [ ] Vitamin and mineral supplement
- [ ] No supplement
- [ ] Other

49.a. If you selected Other, please specify:

Page 8: Section 7 - Hoof Care for Your Horse or Pony

50. During the **past six months** who has most often trimmed your horse's hooves?

- Barefoot trimmer
- Farrier
- Horse's feet not trimmed
- Yourself
- Other

51. During **past six months** have you had your horse shod for any of the following reasons? (Please tick those applicable);

- Brittle hooves
- Club foot
- Cracked hooves
- For competition
- Laminitic hooves
- Low heels
- Navicular syndrome
- Soft hooves
- Riding on hard surfaces
- Thin soles
- I don’t shoe my horse
- I always keep my horse shod
- Prefer Not to Say
- Other

51.a. If you selected Other, please specify:

Page 9: Section 8 – Health Issues

52. During the **past six months** has your horse shown any symptoms of the following **health issues**? (Please tick those applicable):

- ☐ Arthritis
- ☐ Bowed tendon
- ☐ Back problems
- ☐ Colic
- ☐ Dental problems
- ☐ Equine flu
- ☐ Gastric ulcers
- ☐ Hoof abscess
- ☐ Injury which required veterinary attention
- ☐ Laminitis – Acute
- ☐ Laminitis – Chronic
- ☐ Lameness
- ☐ Strangles
- ☐ Thrush
- ☐ No health issues in the last 6 months
- ☐ Prefer not to say
- ☐ Other

52.a. If you selected Other, please specify:

53. During the **past six months** have you had to call the vet out for any **health issues**? *Optional*

- Yes
- No
- Prefer not to say

53.a. If you wish, please tell us if during the **past six months** your horse had any **health issues** which required you to call the vet out for;

54. During the **past six months** have you had made an insurance claim for your horse for any **health issues**?

- Yes
- N/A don't have insurance
- No
- Prefer not to say

54.a. If you wish, please tell us **during the past six months** which **health issues** you made an insurance claim for:

55. During the **past six months** has your horse shown any symptoms of the following **behavioural issues**? (Please tick those applicable):

- ☐ Crib biting
- ☐ Chew or tears rugs (in stall)
- ☐ Difficult with the farrier or trimmer
- ☐ Difficult to lead or turnout
- ☐ Drinks water excessively
- ☐ Eats bedding
- ☐ Pins back ears or lunges out towards people at feeding time
- ☐ ‘Pulls’ faces when people approach or walk by stable
- ☐ ‘Pulls’ faces or fidgets when being tacked up
- ☐ Repetitively licks objects i.e. stall wall
- ☐ Repeatedly kicks the stall wall/door
- ☐ Shows aggression to other horses
- ☐ Shows aggression to people
- ☐ Turns away when people enter the stall
- ☐ Tries to bite or kick when being groomed
- ☐ Tries to kick or bite when people enter the stall
- ☐ Tries to bite or kick when being tacked up
- ☐ Weaves
- ☐ Wind sucking
- ☐ Wood chewing in stall or on fence
- ☐ No behavioural issues
- ☐ Other

55.a. If you selected Other, please specify:

____

56. During the **past six months** have you had made an insurance claim for your horse for any **behavioural issues**?

- Yes
- No
- No insurance n/a
- Prefer not to say

56.a. If you wish, please tell us **during the past six months** which **behavioural issues** you made an insurance claim for:

57. During the **past six months** have you used the services of any of the following equine professionals for your horse?

- Acupuncturist
- Coach or riding instructor
- Equine dental technician
- Equine chiropractor
- Equine nutritional specialist
- Feed company representative
- Equine massage therapist
- Equine physiotherapist
- Equine behavioural professional
- None
- Other

57.a. If you selected Other, please specify:

Page 10: Section 9: Information Sources

58. Please rank from 1 (Always) – 5 (Never) where you most commonly seek information regarding **health** issues affecting your horse.

	Always	Often	Sometimes	Infrequently	Never
Books	○	○	○	○	○
Coach or riding instructor	○	○	○	○	○
Family and friends	○	○	○	○	○
Hoof care practitioner	○	○	○	○	○
Internet websites	○	○	○	○	○
Internet blogs	○	○	○	○	○
Magazines	○	○	○	○	○
Scientific Literature	○	○	○	○	○
Veterinarian	○	○	○	○	○

59. Please rank from 1 (Always) – 5 (Never) where you most commonly seek information regarding **behavioural** issues affecting your horse.

	Always	Often	Sometimes	Infrequently	Never
Books	○	○	○	○	○
Coach or riding instructor	○	○	○	○	○
Family and friends	○	○	○	○	○
Hoof care practitioner	○	○	○	○	○
Internet websites	○	○	○	○	○
Internet blogs	○	○	○	○	○
Equine behavioural professional	○	○	○	○	○
Magazines	○	○	○	○	○
Scientific Literature	○	○	○	○	○
Veterinarian	○	○	○	○	○

60. Please rank from 1 (Always) – 5 (Never) where you most commonly seek information regarding **stable management**.

	Always	Often	Sometimes	Infrequently	Never
Books	○	○	○	○	○
Coach or riding instructor	○	○	○	○	○
Family and friends	○	○	○	○	○
Hoof care practitioner	○	○	○	○	○
Internet websites	○	○	○	○	○
Internet blogs	○	○	○	○	○
Magazines	○	○	○	○	○
Scientific Literature	○	○	○	○	○
Veterinarian	○	○	○	○	○

Page 11: Final Section - Any Additional Comments?

61. Many thanks for your participation in this survey. If you have any additional comments about any of the questions asked or content please add them below.

Page 12: Thank you for your participation.
