# Supplementary material for: Friends, forage, freedom: A cluster analysis investigating horse management styles and welfare in the UK and Ireland
Source: Anim Welf. 2026 Feb 24;35:e15. doi: 10.1017/awf.2026.10073 (PMC12936806; doi:10.1017/awf.2026.10073)
Supplement: Watson et al. supplementary material 2 — Watson et al. supplementary material [file S0962728626100736sup002.pdf]

Friends, forage, freedom: A cluster analysis investigating horse management styles and welfare in the UK and Ireland: Supplementary Material 2

Wendy L Watson https://orcid.org/0009-0005-7178-234X[1], Jill RD MacKayhttps://orcid.org/0000-0001-7134-4829[1], Cathy M Dwyer[2]

[1] The Royal (Dick) School of Veterinary Studies and The Roslin Institute, Easter Bush Campus EH25 9RG, UK

[2] School of Veterinary Medicine and BioSciences, SRUC, Peter Wilson Building, King's Buildings, Edinburgh EH9 3JG, UK

Author for correspondence: Wendy L Watson, email: wendy.watson@ed.ac.uk

Table S1. Horse management questions from the survey highlighting friends (social interaction), forage and freedom (unrestricted movement), which were used to formulate the clusters (n = 13)

**Horse Management Questions from the Survey – Social Interaction n = 4**
Q. 32 - During the past week, if your horse is kept in a stall in a barn, how many other horses can it see from its stall?<br>Q. 38 - On an average day in the past week, how many other horses can your horse interact with freely (i.e., make physical contact with) with when turned out?<br>Q. 43 - On an average day in the past week was your horse most commonly turned out with the same group of horses?<br>Q. 44 - How often does the group of horses turned out with your horse change?
**Horse Management Questions from the Survey – Forage n = 3**
Q. 39 - During the past week, on average day, what type of area was your horse (most commonly) turned out on? (Multiple responses)<br>Q. 46 - During the past week, on an average day, which of the following forage sources did your horse (most commonly) have access to? (Multiple responses)<br>Q. 47 - During the past week, on an average day, how many hours did your horse (most commonly) have access to a forage source?

Horse Management Questions from the Survey – Unrestricted Movement n = 4
Q. 31 - During the past 3 months what type of stall was your horse kept in for the majority of the time?
Q. 38 - During the past week, on average day, how many hours (most commonly) was your horse turned out for each day?
Q. 40 - During the past week what was the size of the area your horse was (most commonly) turned out on?
Q. 41 - During the past month what type of shelter has been most commonly available for your horse when it was turned out?

Table S2. General management questions (demographics) which were used to formulate the clusters (n = 7)

Demographic questions from the survey
Q. 10 - How many years of experience do you have with managing horses or ponies?
Q. 13 - Do you derive an income from equine related activities?
Q. 21 - Do you own this horse? [The horse you reported on in the survey]
Q. 22 - How long have you managed this horse (regardless of whether or not you own it?)
Q. 23 - How long has your horse or pony been in its current location?
Q. 30 - Do you insure your horse or pony for vet expenses?
Q. 51 - During past six months have you had your horse shod for any of the following reasons?

Table S3. Definitions of new groups of variables and the constituent categories (see full survey Supplementary Material 1) that were re-allocated to the new groups to facilitate analysis

Reported Health Issue	Re-grouped As
Strangles/equine flu	Infectious diseases
Lameness, laminitis (acute and chronic), bowed tendon, arthritis	Lameness
Abscess and thrush	Hoof problems
Colic, gastric ulcers	Gastrointestinal issues (GI issues)

Behavioural Issues	Re-grouped As
Crib biting, chew or tears rugs (in stall), drinks water excessively, eats bedding, wind sucking, repetitively licks objects i.e., stall wall, wood chewing in stall or on fence	Abnormal oral behaviours
Difficult with the farrier or trimmer, difficult to lead or turn-out, 'pulls' faces or fidgets when being tacked up, tries to bite or kick when being groomed, tries to bite or kick when being tacked up	Handling issues
Pins back ears or lunges out towards people at feeding time, 'pulls' faces when people approach or walk by stable, repeatedly kicks	Antisocial behaviours

the stall wall/door, shows aggression to other horses, shows aggression to people, turns away when people enter the stall	
Weaving	Weaving

Table S4. Demographic information for the larger survey population (n = 1,501) of respondents in count and percentages. Percentages rounded to 2 decimal places.

**Age**		
Median		45 years
Interquartile Range		21 years
Min		16 years
Max		83 years
**Gender**	**Number of Respondents**	**Percentage of Total**
Female	1475	98.27%
Male	24	1.60%
Prefer Not to Say	2	0.133%
**Country (Respondents)**		
England	836	55.70%
Scotland	433	28.85%
Republic of Ireland	125	8.33%
Wales	59	3.93%
Northern Ireland	45	3.0%
Isle of Man	3	0.20%
**Level of Education**		
Bachelors / Ordinary Degree / Graduate Diploma Honours Degree	478	31.85%
High school degrees or equivalent e.g., GCSEs/National 5s/Standard Grades/Highers	328	21.85%
Master's Degree / Postgraduate Diploma or Certificate	336	22.39%
Modern Apprenticeship/Higher National Certificate/ Higher National Diploma	255	16.99%
PhD	59	3.93%
Other	25	1.67%
No Qualification	20	1.33%
**Total Household Income (Before Tax)**		
Prefer not to say	324	21.59%
£50,000 to £74,999	262	17.46%
£20,000 to £34,999	253	16.86%
£35,000 to £49,999	241	16.06%
£75,000 to £99,999	139	9.26%
Over £100,000	141	9.39%
Less than £20,000	141	9.39%
**Income Derived from Equine Related Activities**		
No not at all	1,123	74.82%
Yes, partially	262	17.46%
Yes, entirely	97	6.46%
Prefer not to say	19	1.27%

Years of Experience Managing Horses		
14 Plus Years	1,197	79.75%
5-14 Years	239	15.92%
Less Than 2 Years-5 Years	61	4.06%
Prefer Not to Say	4	0.27%
**Currently Managing the Horse or Pony (Identified in the Survey)**		
Yes	1,464	97.53%
No	32	2.13%
Don't Know	5	0.33%
**Horse/Pony Owned or Loaned (Identified in the Survey)**		
Yes, I own this horse	1,410	93.94%
Don't own this horse but financially responsible for this horse	37	2.47%
No, I don't own this horse	18	1.20%
Part loan (responsible for the upkeep of the horse)	17	1.13%
No, I don't own this horse but I have some financial responsibility for this horse	12	0.80%
Other	4	0.27%
Missing	3	0.20%

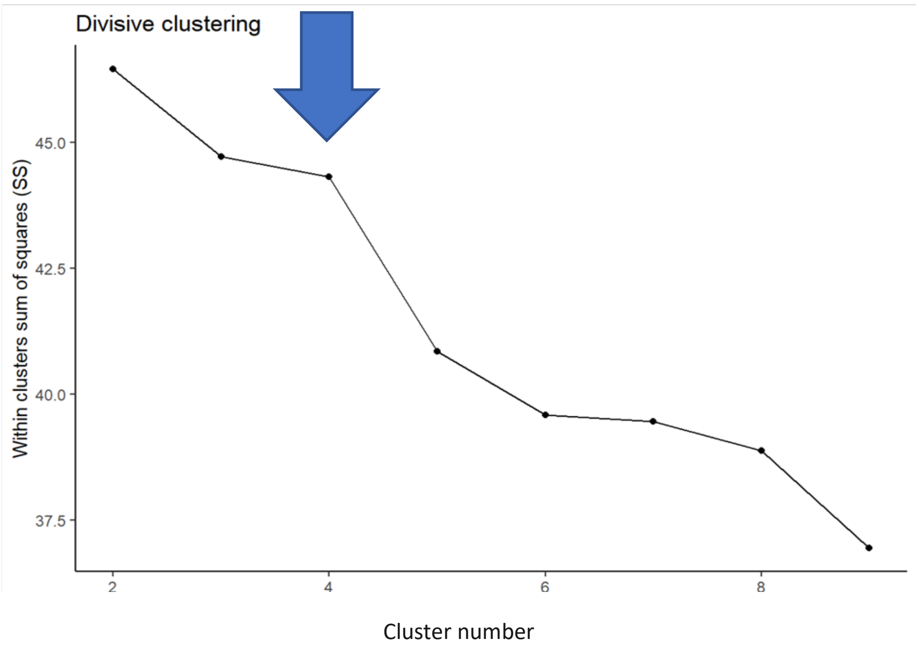

Figure S1. Elbow chart to demonstrate cluster sum of squares 'drop off' at cluster number of 3 (from R) which demonstrates why the number of 3 clusters were chosen
